# Reactive surveillance methods used for malaria elimination in Asia and the Pacific: Results from a 12 country survey

**DOI:** 10.1186/1475-2875-11-S1-P90

**Published:** 2012-10-15

**Authors:** Gawrie NL Galappaththy, Cara Smith Gueye, Kelly Sanders, Christina Rundi, Lasse Vestergaard, Chris Cotter, Roly Gosling

**Affiliations:** 1 Anti-Malaria Campaign, Ministry of Health, Sri Lanka; 2 Global Health Group, University of California, San Francisco, USA; 3 Asia Pacific Malaria Elimination Network, Joint Secretariat: University of Queensland, Australia and the UCSF Global Health Group, USA, Disease Control Division, Ministry of Health, Malaysia; 4 World Health Organization, Philippines

## Background

Moving from malaria control to elimination requires a strong surveillance system, one able to detect all malaria infections, including those without symptoms. Active case detection is designed to do this. One such active surveillance method is reactive case detection. Reactive case detection is the process that a malaria control program undertakes in response to a confirmed case of locally transmitted infection or an imported case that is found in a receptive area. The goal is to find additional cases of malaria infection and halt transmission through treatment of cases and targeted vector control. Although recommended as a tool for malaria elimination, there is little guidance on how a reactive case detection strategy should be implemented, nor is there substantial evidence to guide programs in what type of strategy might work in different epidemiological settings. The Asia Pacific Malaria Elimination Network, or APMEN, is a regional group of 12 country partners with a goal of malaria elimination. One of the main objectives of APMEN is to build the evidence base on malaria elimination, of which active surveillance methods are an important component. This survey aims to provide information on the different strategies in use and will help form the foundation for future studies on reactive case detection in the Asia Pacific.

## Materials and methods

A survey was developed to identify the strategies employed by countries in the areas of: index case investigation, additional screening measures taken in response to a locally transmitted case, training & monitoring of surveillance officers in these activities, reporting structures, SOPs, and additional vector control or entomological surveillance measures used. Analysis of the survey was conducted in Excel by identifying the proportion of positive responses for each question. Some survey respondents required follow up for clarification.

## Results

Nine of 12 countries responded to this survey, all of which were part of the Asia Pacific Malaria Elimination Network. Preliminary results are presented here. The majority of respondents report that any case triggers a case investigation. All countries report that a visit to the index case is conducted as part of the case investigation, but the other measures used vary greatly from country to country. For example, only four re-test the index case while eight supervise treatment adherence. Most countries (from six to nine) report collecting similar information on the index case, such as place of work or travel history, and they map the residence. Only two collect information on history of G6PD deficiency. While nearly all countries (8) define an imported case as those originating outside of the country, the determination of an imported case varies. Eight countries also collect information on internal importation of malaria. Concerning when additional screening measures are used, five countries report additional screening is triggered by a single confirmed case. However, different boundaries for screening are used and different population groups are targeted - only symptomatic or those with and without symptoms, and different distances from the index case. Different triggers are also found for vector control measures and the scale of those measures in communities.

## Conclusions

All countries in the survey employ reactive case investigation, although the scale of this intervention is different for all countries. Also different is the approach of the index case, the additional screening measures, and whether vector measures are employed as part of the procedures. Countries employ these methods without a rigorous evaluation of their effectiveness and without assessing the cost of measures per newly identified case. In order to make evidence-based decisions about surveillance measures, there needs to be more detailed, context-specific guidance on the most effective reactive case detection methods and subsequent screening and vector control.

